# Clinicians’ discretion to contact patients’ at‐risk relatives about their genetic risk: new guidance from Australia's privacy regulator provides timely clarification

**DOI:** 10.5694/mja2.52712

**Published:** 2025-07-13

**Authors:** Jane Tiller, Margaret FA Otlowski

**Affiliations:** ^1^ Monash University Melbourne VIC; ^2^ University of Tasmania Hobart TAS

**Keywords:** Genetic counseling, Genetic testing, Privacy, Prevention and control, Risk management, Medicolegal, Informed consent

Genetic risk information is relevant not just for individuals who are tested, but also for their blood relatives. Cascade genetic testing of at‐risk relatives can save lives. For younger relatives who can access preventive measures, ensuring they know about the availability of testing is particularly important. Challenges with family communication pose a major barrier to family risk notification.^1^ Internationally, assisting index cases to notify their at‐risk relatives is considered a public health imperative.^1^, ^2^, ^3^

One strategy to increase access to cascade screening is for clinicians to contact at‐risk relatives directly, with patient consent. Although international studies indicate this strategy increases cascade testing uptake, its legality has been queried in numerous jurisdictions.^4^, ^5^, ^6^

Following author JT's engagement with the Office of the Australian Information Commissioner (OAIC) — Australia's privacy regulator — regarding the uncertainty about this practice in Australia, the OAIC recently updated its guidance to clinicians about the application of federal privacy law to this question. This guidance clarifies clinicians’ discretion to assist patients with notifying their relatives about genetic risk without breaching federal privacy laws.

## The importance of informing relatives of genetic risk

The importance of ensuring at‐risk relatives can be adequately informed of serious genetic risk was considered by the Essentially Yours inquiry into the protection of human genetic information more than 20 years ago.^7^ At the time, the inquiry Committee was concerned that “privacy legislation inappropriately constrains health professionals’ decisions about [disclosures of genetic information to genetic relatives]” (at 21.83), even where patients do not consent to disclosure, which the Committee concluded should be permissible in certain circumstances.

In 2003, the Committee recommended amendments to the *Privacy Act 1988* (Cth) (Privacy Act) to permit clinicians to disclose patients’ genetic information to their relatives, where it is necessary to lessen or prevent a serious threat to the relative's life, health or safety (recommendation 21‐1), and the development of guidelines for clinicians (recommendation 21‐2). In response, the Privacy Act was amended and the National Health and Medical Research Council developed comprehensive guidelines (section 95AA guidelines)^8^ for clinicians to follow when patients do not consent to disclosure.^9^, ^10^

Under these guidelines, Dr Netix in our hypothetical case study (Box 1) could legally notify Cassie's sister Molly of her possible genetic risk, even without Cassie's consent. However, Australian clinicians have a poor understanding of this discretion,^11^ leading to significant confusion about their ability to assist patients by directly notifying at‐risk relatives with patient consent.

## Notifying at‐risk relatives with patient consent

It seems clear on its face that if clinicians can legally notify patients’ relatives of their possible genetic risk even without the patients’ consent, this should also be legal where the patient does consent.^12^ However, Australian clinicians continue to express concerns that using the relatives’ contact details to notify them might breach the relatives’ privacy.^13^

The Essentially Yours Committee's consideration^7^ presumed that no issues arose where patients consented to disclosure to their relatives, and directed their recommendations towards ensuring that notification could occur even without patients’ consent. Although the s95AA guidelines were developed primarily for this circumstance, they also carry this underlying presumption. Section 3.4.3, titled “Process of cascade contact”, describes how cascade contact of at‐risk relatives proceeds where consent is given by the patient, stating:

… a step‐by‐step process of cascade contact allows more genetic relatives to receive information about a genetic condition. Each genetic relative who is notified about their increased risk and makes contact with the disclosing health practitioner is asked for consent to contact his or her genetic relatives.^8^

## Do patients and the general public want direct notification of genetic risk?

During the same period as the Essentially Yours Inquiry, the South Australian clinical genetics service conducted a randomised study from 2001 to 2004 (with ethics approval) to determine whether directly contacting relatives would increase the uptake of cascade testing.^14^ In the cohort where relatives were contacted directly, the proportion of relatives who undertook cascade testing almost doubled. This increase in cascade testing uptake in directly contacted cohorts has been replicated internationally.^15^ The South Australian service continued with direct contact of relatives until mid‐2019, when they reduced this practice to specific patient request scenarios only, due to resource limitations.^16^

We recently surveyed South Australian patients — both those who were directly contacted and those who were not — finding strong support for direct contact and few privacy concerns.^16^ Similarly, our recent survey of 1030 Australians from the general public found that people overwhelmingly want to be told about the genetic risk of medically actionable conditions, prefer to be notified by clinicians, and have few privacy concerns about this practice.^17^ It is clear that the Australian public and patient populations want to be told about their genetic risk, and are generally comfortable with clinicians collecting and using relatives’ contact details from patients to notify them.

## What does the new guidance say?

The OAIC maintains a *Guide to health privacy*.^18^ Chapter 8 of the guide relates to using and disclosing genetic information in case of a serious threat. Previous versions of Chapter 8 have focused on the discretion available under the s95AA guidelines to disclose genetic information to at‐risk relatives without patient consent.

In 2024, following years of research and public discussion, JT sought advice from the OAIC regarding the application of the Privacy Act to the notification of at‐risk relatives with patient consent. The OAIC recognised the clinical importance of this issue, and in May 2025, it updated Chapter 8 to clarify that clinicians may legally collect relatives’ contact details from patients and use those to contact at‐risk relatives with patient consent.^18^ The updates made no changes to the current guidance about notification without patient consent. The guidance makes it clear that the collection and use of the relative's contact details must still be done in accordance with the Privacy Act, but are permitted where a clinician reasonably believes the collection and use are necessary to lessen or prevent a serious threat to the life, health or safety of that relative.^18^

The Privacy Act requires that certain types of personal information are only collected or used with the consent of the person they belong to, unless it is unreasonable or impracticable to obtain consent (s16A (1)). The OAIC's updated Chapter 8 guidance also clarifies that “it is likely to be impracticable to seek a relative's prior consent to collection or use of their contact details, as the health professional will not know about the relative other than through the patient and cannot contact the relative without collecting the contact details from the patient”.^18^ For the avoidance of doubt, the updated guidance further confirms that, “once you have collected the contact details, you can use them to contact the relative to inform them of their possible genetic risk, as this is the primary purpose for which you collected the information”. The guidance advises that even if a clinician already holds a relative's contact details in their records (eg, as a next of kin), they can use them for the secondary purpose of informing that person of their genetic risk, if they are satisfied that it is unreasonable or impracticable to obtain consent. Chapter 8 also confirms that a genetic variant that increases the risk of developing a certain cancer may be a serious threat that can be lessened or prevented by disclosing the threat to the relative.

This updated guidance makes it clear that, in the case study presented (Box 1), Dr Netix could collect Molly's contact details from Cassie, and use them with Cassie's consent to notify Molly of her possible genetic risk. It also includes a flow chart to assist clinicians in applying this discretion (Box 2).^18^ It should be noted that the federal Privacy Act applies to private health providers, but state and territory public hospital staff are subject to local privacy laws and regulations. However, our previously published privacy analysis^12^ concludes that direct notification can be conducted in accordance with the regulations in each state and territory, following the same principles set out in the OAIC's updated guidance. Direct consideration of this question by state and territory regulators will assist clinicians further.

Box 1 Case study*

**Table:** 

Cassie is a young woman with a *BRCA1* genetic variant, which significantly increases her risk of developing breast and ovarian cancer. Cassie's first‐degree relatives each have a 50% risk of also having the *BRCA1* variant and should be offered genetic testing and preventive care.
Cassie has a strained relationship with her sister Molly, who is unaware of her risk and the opportunity to have genetic testing. Cassie does not want to contact Molly but believes she should be notified about her risk. Cassie asks her clinician, Dr G Netix, to write to Molly directly to give her this information and provides Molly's contact details. Dr Netix is unsure whether he is legally permitted to use Molly's contact details to notify her of her genetic risk, or whether this is a breach of privacy laws.

* Not a real patient; names are fictional.

Box 2 **Flow chart developed by the Office of the Australian Information Commissioner to guide clinicians on contacting at‐risk relatives of patients**

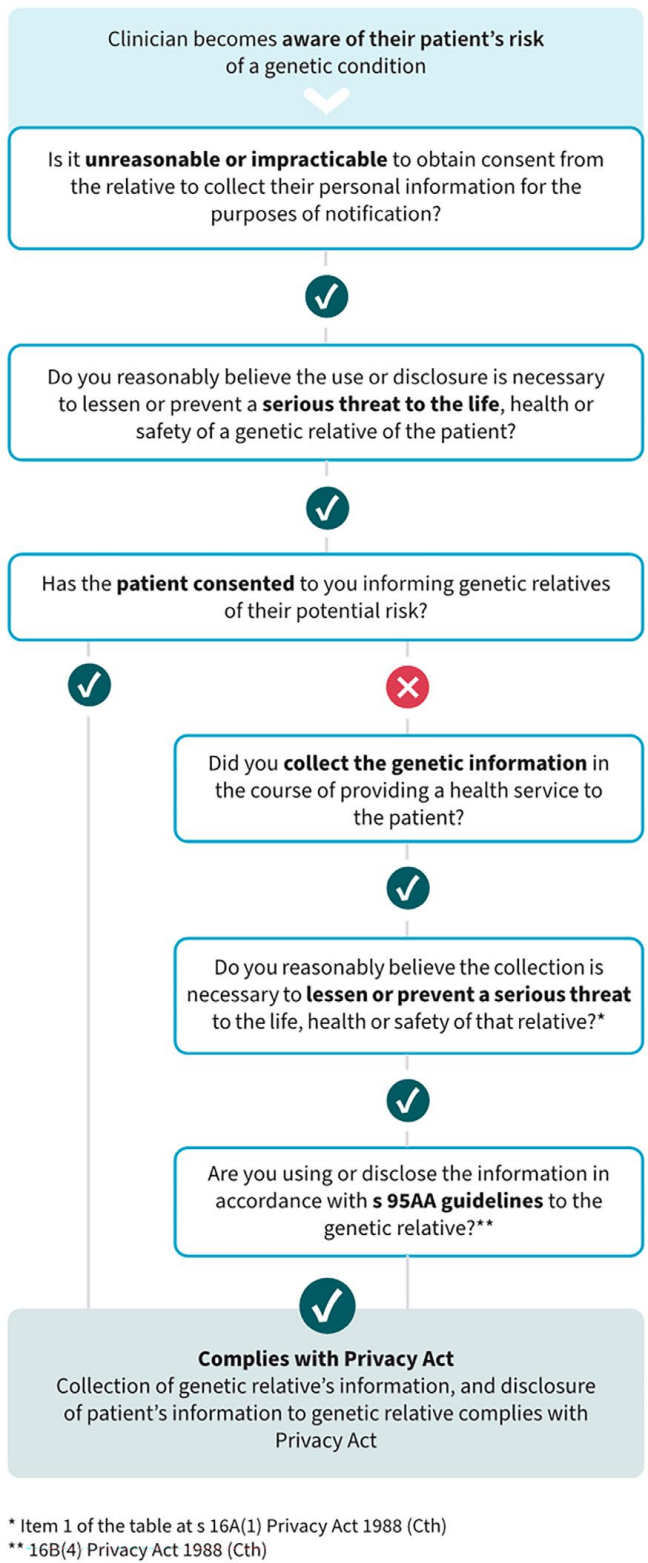

Source: Figure reproduced with permission from the Office of the Australian Information Commissioner.^18^

## What about the right not to know?

One of the primary ethical arguments sometimes raised against notifying relatives directly is the amorphous “right not to know”. This is not a right enshrined in privacy legislation, but an ethical overlay that is prudent to consider when contemplating the disclosure of genetic information. There are two important points to note here. The first is that the current practice employed to assist with notification of relatives in Australia is the provision to patients of a “family letter” to share with their at‐risk relatives.^13^ This practice does not give any greater consideration to the “right not to know” than direct contact methods do — in the context of patient consent, both methods rely on the patient to decide whether relatives should be notified, and only the method of delivery of the letter (via the patient or directly from the clinician) differs.

The second point is that there are numerous types of genetic risk information. Risk information can be serious or mild, and can be actionable or non‐actionable. For the provisions discussed here to apply, the risk must be serious, and it must be able to be lessened or prevented by disclosing that threat to the relative. This matter was considered previously by the Essentially Yours report, stating (at 21.86):

Another concern relates to the need to recognise that some people may not wish to know about their genetic risk. However, if the circumstances in which disclosure is permitted are limited to situations where it is necessary to lessen or prevent a serious risk, it is reasonable to assume that only rarely would individuals not wish to know about the risk.^7^

## Discretion versus duty

Some clinicians express concerns about the potential for a “duty to warn” or obligation to be implied in this context,^6^ and that this may create resourcing implications.^13^ The guidance makes it clear that it “is not intended to imply the existence of an obligation for health service providers to identify and contact all relatives who may be at high risk of having a genetic predisposition, but is aimed at clarifying how the Privacy Act applies to providers who choose to do so”.^18^

## What next?

When surveyed, the majority of representatives from Australian genetics services agreed that a clinical guideline would assist them to understand their discretion in this area.^13^ Some guidance on issues such as identifying whether the serious threat threshold is met is available from the guidelines developed in the context of notification without patient consent.^8^ Now that the OAIC has clarified that relatives’ contact details can be collected from patients and used to notify them about their genetic risk, without breaching the Privacy Act, the development of a clinical guideline to assist clinicians would be timely. Consideration and guidance from privacy regulators in each state and territory about the interpretation of local laws would assist with this.

## Author contributions

Tiller J: Conceptualization; writing – original draft. Otlowski MFA: Supervision; writing – review and editing.

## Open access

Open access publishing facilitated by Monash University, as part of the Wiley – Monash University agreement via the Council of Australian University Librarians.

## Competing interests

No relevant disclosures.

## Provenance

Not commissioned; externally peer reviewed.
